# AUV-Aided Optical—Acoustic Hybrid Data Collection Based on Deep Reinforcement Learning

**DOI:** 10.3390/s23020578

**Published:** 2023-01-04

**Authors:** Fanfeng Bu, Hanjiang Luo, Saisai Ma, Xiang Li, Rukhsana Ruby, Guangjie Han

**Affiliations:** 1 College of Computer Science and Engineering, Shandong University of Science and Technology, Qingdao 266590, China; 2 College of Computer Science and Software Engineering, Shenzhen University, Shenzhen 518060, China; 3 Department of Information and Communication Systems, Hohai University, Changzhou 213022, China

**Keywords:** autonomous underwater vehicles, optical–acoustic multi-modal communication, data collection, path planning, deep reinforcement learning

## Abstract

Autonomous underwater vehicles (AUVs)-assisted mobile data collection in underwater wireless sensor networks (UWSNs) has received significant attention because of their mobility and flexibility. To satisfy the increasing demand of diverse application requirements for underwater data collection, such as time-sensitive data freshness, emergency event security as well as energy efficiency, in this paper, we propose a novel multi-modal AUV-assisted data collection scheme which integrates both acoustic and optical technologies and takes advantage of their complementary strengths in terms of communication distance and data rate. In this scheme, we consider the age of information (AoI) of the data packet, node transmission energy as well as energy consumption of the AUV movement, and we make a trade-off between them to retrieve data in a timely and reliable manner. To optimize these, we leverage a deep reinforcement learning (DRL) approach to find the optimal motion trajectory of AUV by selecting the suitable communication options. In addition to that, we also design an optimal angle steering algorithm for AUV navigation under different communication scenarios to reduce energy consumption further. We conduct extensive simulations to verify the effectiveness of the proposed scheme, and the results show that the proposed scheme can significantly reduce the weighted sum of AoI as well as energy consumption.

## 1. Introduction

Accompanied by the increasing demand for ocean exploration and protection, underwater sensor networks (UWSNs) have received more attention as these play an important role in diverse marine applications, such as coastal monitoring and protection, marine resource exploration, disaster warning and military operations [1,2,3,4]. However, due to the harsh hydrographic and geographical environment, it is difficult to collect data from underwater sensor devices via a long-range routing path. Even if the monitored data can be transmitted through multi-hop routing technologies, there may be heavy workload near the sink with extra energy consumption [5]. Furthermore, as the battery power of underwater sensor nodes is severely limited and difficult to be recharged underwater, it is not energy-efficient to upload large volume of ocean monitoring data to the sink directly. Moreover, with marine security operations, it is better to collect secret data nearby the monitoring sensors. To solve the aforementioned problems, autonomous underwater vehicles (AUVs) have been rapidly developed in recent years in terms of data storage and signal processing capabilities, which can better enable underwater mobile data collection. Moreover, the durability and mobility of AUVs alleviate the unbalanced energy consumption problem of underwater sensors [6,7].

To collect data in an efficient manner, various underwater communication technologies have been investigated, such as acoustic and optics [8]. Currently, although underwater acoustic communication (UAC) has become the most widely used technology due to its unique advantages (e.g., long-range communication), it is limited by its shortcomings (e.g., low bandwidth, slow speed, high bit error rate and large delay) [9]. To address these issues, underwater optical communication (UOC) has emerged as an alternative solution, as it has a higher propagation speed (2.255 $\times 10^{8}$ m/s) and higher data rate (up to hundreds of Mbit/s) over short to medium-range transmissions [10,11]. As both acoustic and optical communication have their pros and cons, employing multi-modal underwater communication systems in UWSNs has become a potential approach to improve network performance [12,13].

To facilitate mobile data collection in such multi-modal networks, it is necessary to satisfy the varying requirements of marine applications by combining the potential advantages of AUVs [14]. Combined multi-modal data collection via AUV is divided into two categories, such as acoustic multi-modal and acoustic-optical multi-modal. In acoustic multi-modal data collection, the sensor node transmits control information using low-frequency acoustic waves and guides the AUV to the designated area, and then, it switches to a high-frequency UAC modem to transmit the data [15]. In this case, the high energy consumption of the UAC shortens the lifetime of the sensor node when transmitting large volume of network data. Whereas, in the acoustic–optical multi-modal data collection, the UAC provides the capability for the AUV to approach the sensor node through long-range guidance and assists with alignment for the optical communication. The subsequent proximity of the UOC data transmission not only improves data transmission efficiency but also saves energy for transmission [16]. However, limited by the UOC range, it is necessary for the AUV to move slowly close to the sensor node to build an optical link reliably, which increases traveling time. A promising solution to the above problem is to engage both the UAC and UOC in data collection, such as transmitting a small volume of data over long distances using the UAC and retrieving and offloading large amounts of data using the UOC [17,18].

Although the aforementioned pioneering studies have laid a solid foundation for multi-modal data collection, there are still some issues when applied to mobile data collection. Firstly, the AUV should adaptively select the best communication technology according to the specific marine operational data requirements (e.g., data importance and packet size). For example, high-quality data collection (e.g., high-resolution images with 4K size) with UOC can prolong the network lifetime of the UWSN by sacrificing the energy consumption of the AUV, and when the volume of data is relatively less, UAC can be used for remote collection to reduce the energy consumption and travel time of the AUV. Furthermore, the AUV should complete the data collection operation quickly to guarantee the freshness of data as the data value usually decays over time [19]. Generally, the age of information (AoI) can be used to measure data freshness in mobile data collection scenarios [5,20,21]. By optimizing AoI, the requirement of the network for timely data delivery can better be satisfied. Therefore, in a multi-modal AUV-enabled mobile data collection scheme, how to optimize the trajectories of AUV and select a communication option to minimize both AoI and energy consumption based on the size and the importance of the packets is a critical issue.

To solve the aforementioned issues, in this paper, we propose an acoustic–optical multi-modal mobile data collection scheme. Based on the type and the size of data, the AUV intelligently searches for the optimal trajectory and communication options using the deep reinforcement learning (DRL) approach, thereby minimizing the AoI and extending the lifetime of the sensor network. To the best of our knowledge, this is the first study which focuses on integrating an acoustic–optical multi-modal option with optimal AUV path planning for reliable and timely mobile data collection leveraging the DRL approach. The main contributions of this study are listed as follows.

- We investigate an AUV-assisted underwater trajectory planning problem for data collection by integrating the complementary advantages of both acoustic and optical communication with data diversity to perform reliable and timely mobile data collection.
- We propose a DRL-based AUV-assisted multi-modal mobile data collection scheme in which we consider several key factors, such as data importance, packet size and data collection option, to minimize AoI and reduce energy consumption.
- We propose an optimal angle steering algorithm for AUV navigation to reduce energy consumption, in which the steering angle of the AUV is determined based on the AUV and sensor positions as well as the data collection option.

The rest of the paper is structured as follows. We briefly review the related works in Section 2. In Section 3, we introduce the network model with necessary background. In Section 4, we analyze the problem of the multi-modal data collection. In Section 5, we describe the proposed scheme of DRL-based multi-modal data collection in detail. We evaluate the performance of the proposed scheme in Section 6. Finally, we conclude the paper in Section 7.

## 2. Related Works

In recent years, multi-modal communication has become a research topic to improve network performance and optimize data transmission in various marine application scenarios. Commonly adopted multi-modal technologies include acoustic multi-modal communication and acoustic–optical hybrid communication [13,22,23,24]. Among them, the acoustic multi-modal communication is constructed by a set of UAC modems working on different frequency bands [13]. In [22], the authors proposed a multi-modal underwater routing protocol based on the reinforcement learning technique. In this protocol, the reliability and delay of data transmission are optimized by UAC modems in multiple frequency bands. To explore the advantages of UAC and UOC during data transmission, Shen et al. proposed an acoustic–optical multi-modal routing scheme based on packet size and link adaptation, which reduces packet loss and end-to-end delay [23]. However, the challenge of unbalanced energy consumption still exists in the multi-hop underwater networks.

The AUV-assisted data collection can mitigate the energy consumption unbalance that occurs in multi-hop routing. Han et al. [18] explored the characteristics of underwater acoustic and optical communication in AUV-assisted data collection and showed that hybrid acoustic–optical data collection outperforms the one with a single acoustic modem in terms of both throughput and energy consumption. To cope with the impact of the harsh underwater environment, Luo et al. [25] maximize the network throughput by capturing the dynamic characteristics of the channel and the mobility of the AUV. Hu et al. [17] proposed a mobile data collection method for the heterogeneous sensor network using multi-hop acoustic communication to build an intra-cluster network where CHs collect large-scale data and upload them to a mobile receiver via optical communication. Although the aforementioned schemes improve the efficiency of data collection, these ignore the difference in the importance of data and the decay of freshness over time.

To handle the aforementioned issues, Gjanci et al. [16] proposed a greedy adaptive navigation algorithm to guide AUV for data collection, which considers the characteristics of data decay, but it is only applicable to sparse networks due to the unavoidable long paths. To deliver emergency data faster, Liu et al. [26] proposed a hybrid data collection scheme, in which the urgent data are routed using a multi-hop scheme, while the delay-insensitive data are collected by AUVs. Duan et al. [27] studied a hierarchical data collection problem using AUVs to optimize the information quality of the collected data while considering the importance and timeliness of the events.

More recently, researchers have proposed the concept of AoI to model the timeliness of data while considering the quality of experience (QoE) [28]. Khan et al. [29] provided an optimization algorithm to ensure the freshness of the collected data. Fang et al. [5] used a vocational queuing model to improve the data reliability and peak AoI of the data. Then, the communication link is established using the UAC when the AUV arrives near the node. Al-Habob et al. [30] proposed a framework to optimize the trajectories of AUV and minimize the normalized weighted sum of the average AoI. Wu et al. [31] studied the AUV transmission scheduling policy by considering both the age and the importance of the message.

Although the aforementioned approaches have promoted the study of underwater data collection, only a single communication technology was considered for the data collection. Moreover, none of them addressed the issue of leveraging multiple data types and multiple communication technologies to improve data freshness and energy efficiency. To address this issue, in this paper, we propose a AUV-assisted acoustic and optical multi-modal data collection scheme, in which we use the DRL method to optimize AUV trajectories, AoI and energy consumption by considering different communication options and packet size.

## 3. Network Model

### 3.1. Network Architecture

**Figure 1:**
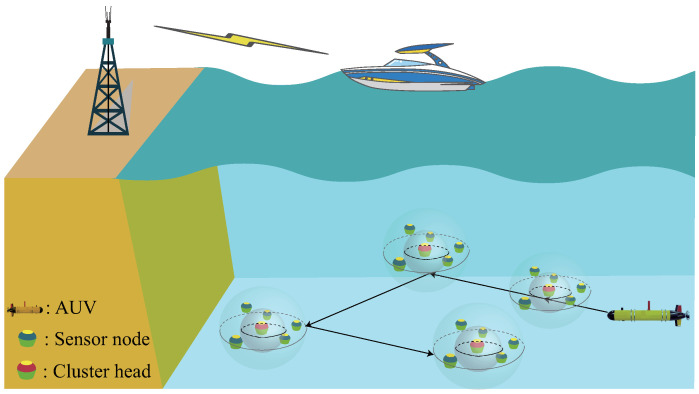
Illustration of the underwater network model.

As shown in Figure 1, we consider an AUV-based multi-modal data collection network where the deployed nodes are classified into ordinary nodes $S=\{s_{1},s_{2},\ldots ,s_{M}\}$, cluster heads $CHs=\{c_{1},c_{2},\ldots ,c_{N}\}$ and sink node according to their different functions. The sensor nodes are statically deployed on the seabed using anchor chains, where the locations of the nodes are assumed to be known. The CHs perform intra-cluster data fusion and data compression [27] and then wait for the AUV to arrive and collect the data. In particular, during the network formation phase, all sensor nodes are divided into multiple clusters based on spatial distance, and only one node in each cluster is selected as the CH, while other nodes are used as ordinary nodes for data collection [32]. The AUV performs global data collection around all CH nodes and finally reports the data to the sink node.

In the multi-modal network, each node is equipped with both UAC and UOC modems for multi-modal communication, and they have the same initial energy, sensing and communication capabilities. Specifically, it includes an acoustic modem for exchanging data at a low transmission rate over a long distance and an optical modem with a relatively short transmission distance and high data rate [33]. Meanwhile, the AUV has similar communication capability to ensure the data transmission [16]. Without loss of generality, we assume that the data arrival rate of sensor node obeys a Poisson random distribution with parameter λ. When the AUV visits $c_{i}$, the CHs package the sampled data block into the packets of length $B_{i}$ with timestamp $T_{i}$.

### 3.2. Node Clustering Phase

We assume that the nodes are randomly deployed in the target area to monitor the underwater environment, and the nodes are clustered. In the initial phase, the sink nodes know the location of each node and determine the number of clusters based on the network size, and then, the target area is divided equally into several square areas. The sink node broadcasts the subregion message to all nodes, and each node determines its own cluster identifier based on its position. Nodes with the same identifier belong to the same cluster [26].

The CHs should be selected for inter-cluster data collection and communication with the AUV. The selection of CH is carried out according to the procedure as follows. The number of optical and acoustic communication neighbors of each node in the sub-region is first obtained, and then, the node with the highest number of optical communication neighbors and the remaining energy satisfying the energy threshold requirement is selected as the CH. Then, the above operation is repeated until all CHs in the target region are determined. Finally, a confirmation packet is sent by the sink to the designated CHs. At the end of the data collection process, all CHs are evaluated, and when the energy of the CH is less than the energy threshold, the network performs a new CH selection round.

### 3.3. Acoustic Data Collection Link

When the AUV traverses near the node, it is necessary to construct a communication link for data collection. As for the acoustic link, the acoustic wave is affected by the absorption of medium and the scattering of impurities in water. The path loss of underwater acoustic channels is related to frequency *f* and distance $d_{ac}$. To this end, the total attenuation is given as follows [34].

(1) $$A\left(d_{ac},f\right)=d_{ac}^{k}a{\left(f\right)}^{d_{ac}},$$

where *k* = 1.5 represents propagation loss, and a(f) is the absorption coefficient in dB/km given by the Thorp formula [35]

(2) $$\begin{array}{c} 10\log a\left(f\right)=0.11\frac{f^{2}}{1+f^{2}}+44\frac{f^{2}}{4100+f^{2}}\\ +2.75\times 10^{-4}f^{2}+0.003. \end{array}$$

Consequently, given the acoustic signal transmit power $P_{trans}^{ac}$ and frequency *f*, the signal-to-noise ratio (SNR) can be expressed as [36]

(3) $${SNR}_{ac}\left(d_{ac},f\right)=\frac{P_{trans}^{ac}/A\left(d_{ac},f\right)}{N\left(f\right)\Delta f},$$

where N(f) and Δ(f) represent the total noise level including four kinds of interference noise and the bandwidth of the receiver, respectively. Therefore, the transmission power of acoustic communication satisfying the minimum $SNR_{\min}^{ac}$ is expressed as

(4) $$P_{trans}^{ac}=SNR_{\min}^{ac}A\left(d_{ac},f\right)N\left(f\right)\Delta f.$$

### 3.4. Optical Data Collection Link

For the optical link, the path loss PL of the underwater wireless optical link can be expressed as [37]

(5) $$PL\approx 10\log\left({\left(\frac{D_{r}}{2\theta d_{op}}\right)}^{2}e^{-cd_{op}{\left(\frac{D_{r}}{\theta d_{op}}\right)}^{\zeta }}\right),$$

where $D_{r}$ represents the aperture diameter of the receiver and θ denotes half of the transmitter beamwidth, $d_{op}$ represents the distance between transceivers, and *c* and ζ represent the extinction coefficient and turbidity of water quality, respectively. Subject to the optical-to-electric conversion efficiency of the receiver, a minimum received power per bit is defined as $P_{rec}^{op}$. Then, the transmission power of the UOC is expressed as

(6) $$P_{trans}^{op}=\frac{P_{rec}^{op}}{PL}.$$

In order to ensure robust optical communication, it is necessary to control the minimum $SNR_{\min}^{op}$ requirements [38].    

(7) $$SNR_{\min}^{op}={\left[\frac{P_{trans}^{op}e^{-cd_{op}}D_{r}^{2}\cos\varphi }{\left(\tan^{2}\theta \right)4d_{op}^{2}NEP}\right]}^{2},$$

where NEP represents the noise equivalent power and φ is the offset angle between transceivers. According to the Lambert *W* function [39], the maximum underwater optical communication distance while satisfying the communication $SNR_{\min}^{op}$ can be obtained by

(8) $$d_{op}=\frac{2W\left(\frac{c}{4}{\left[\frac{{\left(SNR_{\min}^{op}\right)}^{\frac{1}{2}}NEP\tan^{2}\theta }{P_{trans}^{op}D_{r}^{2}\cos\varphi }\right]}^{-\frac{1}{2}}\right)}{c}.$$

Since optical modems are usually directional, in order to receive optical signals from any direction, we assume that an omni-directional optical modem can be achieved by using multiple LEDs [40].

## 4. Multi-Modal Data Collection Analysis

When performing multi-modal data collection via AUV, the freshness of the collected data and the energy consumption of the network nodes need to be fully considered. The choice of communication options fundamentally affects the data collection efficiency. Among the various communication options, the UOC is capable of transmitting a large volume of data rapidly to reduce transmission latency but increases the navigation time and energy consumption of the AUV. Meanwhile, the UAC has a lower bandwidth but can collect small volume of data over long distances to reduce the travel time of the AUV. Consequently, to collect data in a timely and efficient manner, several key factors, such as the data collection option, data type, packet size and AoI requirement, should be fully analyzed and integrated into the optimal path-planning scheme for data collection.

### 4.1. Problem Analysis

The primary goal of the mobile data collection in this paper is to minimize both the weighted average AoI and the energy consumption. The factors that influence the AoI include the AUV trajectory, the data transfer time and the importance of the data. Consequently, to minimize the weighted average AoI, the optimization problem can be expressed as

(9) $$\begin{array}{cc} \min & \frac{1}{N}\sum_{i=1}^{N}A_{i}+\beta \sum_{i=1}^{N}e_{i}, \end{array}$$

(9a) $$\begin{array}{cc} s.t.\; & x_{i,t}^{ac}\in \left\{0,1\right\}, \end{array}$$

(9b) $$\begin{array}{cc} \; & \;x_{i,t}^{op}\in \left\{0,1\right\}, \end{array}$$

(9c) $$\begin{array}{cc} \; & \sum_{t=1}^{T}x_{n,t}^{ac}+x_{n,t}^{op}=1,\;\forall n\in N \end{array}$$

(9d) $$\begin{array}{cc} \; & \;E_{AUV}<energy_{AUV}, \end{array}$$

(9e) $$\begin{array}{cc} \; & \;P_{0}=\left(x_{0},y_{0}\right), \end{array}$$

where $A_{i}$ denotes the final result of AoI when the data from $c_{i}$ reach the sink node, $e_{i}$ denotes the energy consumption by $c_{i}$ during data collection, and $x_{i,t}^{ac}=1$ indicates that the AUV has reached the acoustic communication range of CH $c_{i}$ and receives data through UAC. Otherwise, $x_{i,t}^{ac}=0$ holds. Similarly, $x_{i,t}^{op}$ represents an optical communication indicator. $E_{AUV}$ is the energy consumption of the AUV in data collection, and $energy_{AUV}$ indicates the initial total energy of the AUV. The constraint in (9c) is used to ensure that each node can select just one communication option during data collection. The constraint in (9d) is to guarantee that the AUV cannot consume all of its energy. Finally, the constraint in (9e) is to determine the initial position of AUV.

The optimization problem (9) is a non-linear integer programming problem, which is intractable due to the presence of binary variables and non-convex objective function. In the following section, we model this as a Markov decision process (MDP) to be solved by leveraging the DRL approach.

### 4.2. Definition of AoI

The AoI is an important metric to portray the freshness of collected data and is defined as the time elapsed between the data collected by the AUV from the CHs until its delivery to the sink node [41]. We use $\delta_{i,t}$ to denote the AoI collected from $c_{i}$ in the navigation trajectory at time *t*. When $t<T_{i}$, the information of CH $c_{i}$ is not sampled since it is not visited, and thus, $\delta_{i,t}=0$ holds. Otherwise, $\delta_{i,t}=t-T_{i}$ holds. Then, the AoI of $c_{i}$ at the start of time slot *t* is given by the following relation.

(10) $$\delta_{i,t}=\left\{\begin{array}{cc} 0, & \mathrm{if}\;\;t<T_{i}\\ t-T_{i}, & \mathrm{otherwise} \end{array}\right..$$

The primary factors affecting AoI during data collection include data transmission delay and AUV sailing time. We use $T_{i}^{ac}$ and $T_{i}^{op}$ to denote the data transmission time using UAC and UOC, respectively. The time to transmit $B_{i}$ bits by the UAC can be written as

(11) $$T_{i}^{ac}=\frac{B_{i}}{R_{ac}}+\frac{d_{i}}{V_{ac}},$$

where $R_{ac}$ and $V_{ac}$ indicate the data rate and transmission velocity of the underwater acoustic modem, respectively. Similarly, the data transmission time of the UOC at data rate $R_{op}$ and transmission velocity $V_{op}$ can be obtained as follows.

(12) $$T_{i}^{op}=\frac{B_{i}}{R_{op}}+\frac{d_{i}}{V_{op}}.$$

To collect the monitored data, the AUV travels from the sink $p_{0}$, collects data from each of the *N* CHs according to a pre-determined trajectory, and then returns to the sink node after completing the task. Assume that the travel trajectory of the AUV $P=\{p_{0},p_{i},\ldots ,p_{j},p_{0}\}$, and thus the travel time of the AUV can be expressed as

(13) $$T_{travel}=\frac{D(P)}{V_{AUV}},$$

where D(P) and $V_{AUV}$ denote the total distance and velocity traveled by the AUV, respectively.

According to (11)–(13), from the moment $T_{i}$ when AUV arrives at CH $c_{i}$ to the moment $T_{i+1}$ when it finishes collecting data and moves to the next data collection point, the AoI of CH $c_{i}$ can be expressed as $T_{i}^{m}+t_{i,i+1}^{travel}$. The optical communication has a much smaller transmission delay compared to the acoustic waves. However, the acoustic communication enables long-range transmission that significantly reduces the travel time of the AUV. The time delay caused by data transmission is mainly determined by the data size $B_{i}$, and so the data collection time and traveling time need to be considered jointly to reduce the decline of data freshness. Then, at moment $t=T_{i+1}$, the AoI collected from CH $c_{i}$ refers to

(14) $$\delta_{i,T_{i+1}}=\left\{\begin{array}{cc} T_{i}^{ac}+t_{i,i+1}^{travel}, & \mathrm{if}\;b_{i}\;=\;1\;\mathrm{and}\;x_{i,t}^{ac}\;=\;1\\ T_{i}^{op}+t_{i,i+1}^{travel}, & \mathrm{if}\;b_{i}\;=\;1\;\mathrm{and}\;x_{i,t}^{op}\;=\;1 \end{array}\right.,$$

where $b_{i}=1,\;i=\left\{1,2,\ldots ,N\right\}$ indicates that the data of CH $c_{i}$ has been collected; otherwise, $b_{i}=0$ holds. When the AUV arrives at the sink node, the AoI of $c_{i}$ is

(15) $$A_{i}=\sum_{k=i}^{N}\eta_{i}\delta_{k,T_{k+1}},$$

where $\eta_{i}$ denotes the importance weight of the data collected by CH $c_{i}$, and $\sum_{i=1}^{N}\eta_{i}=1$. The higher its value, the greater the data importance.

### 4.3. Energy Consumption Associated with Data Collection

To satisfy the energy constraint (9d) in the optimization problem (9), we analyze the AUV energy consumption and node energy consumption. In the data collection process, there are extra costs associated with the AUV if it runs out of energy before returning to the sink node. Therefore, the trajectory of the AUV should be scheduled to minimize energy consumption. The power of the AUV at each time slot mainly consists of the sum of propulsion power $\Phi_{prop}$ and hotel load power $\Phi_{H}$ [42]. The hotel load $\Phi_{H}$ is the power consumed by all subsystems other than propulsion mechanism and is typically negligible in comparison with $\Phi_{prop}$ [43]. Therefore, the power of the AUV trajectory can be expressed as

(16) $$\Phi_{prop}=\frac{\rho }{2\eta_{p}}C_{D}A_{s}{∥V_{AUV}∥}^{3},$$

where $∥\cdot ∥$ denotes the Euclidean vector norm and ρ is the density of water. $\eta_{p}$, $C_{D}$ and $A_{s}$ indicate the efficiency of the AUV’s propulsion system, the drag coefficient and the wetted surface area, respectively [7]. Consequently, with the relations in (4), (6), (11)–(13) and (16), the total energy consumption can be expressed as

(17) $$\begin{array}{cc} E_{tot} & =\sum_{i=1}^{N}e_{i}+\varpi \Phi_{prop}T_{travel}\\ \; & =\sum_{i=1}^{N}x_{i}^{ac}T_{i}^{ac}P_{trans}^{ac}+x_{i}^{op}T_{i}^{op}P_{trans}^{op}+\varpi \Phi_{prop}T_{travel}, \end{array}$$

where ϖ is a weighted parameter that measures the balance between the energy consumption of the sensor node and that of the AUV.

## 5. Proposed DRL-Based Multi-Modal Data Collection Scheme

In this section, we design the AUV multi-modal data collection scheme by leveraging the DRL approach. In this scheme, we first provide the MDP formulation and then present a multi-modal steering angle optimization (MSAO) algorithm for the AUV. Afterwards, we design the AUV path planning using the Deep Q Network (DQN) method for multi-modal mobile data collection.

### 5.1. MDP Formulation

When the network nodes are clustered, the next goal is to find an optimal CHs data collection strategy. The AUV-assisted data collection problem can be formulated as an MDP to be solvable by the DRL approach, which is represented by <S,A,P,R,γ> five tuples. Here, S is the state of the environment, A is the set of actions of the agent, P is the state transition probability, R is the reward function, and γ denotes the discount factor. In particular, at time slot *t*, the agent observes state $s_{t}$ and chooses an action to be performed. Then, the environment state is transferred with probability $p_{s_{t},s_{t+1}}$ to $s_{t+1}$ and the agent obtains a reward $r_{t}$ from the environment. In this paper, the AUV is considered as the agent to collect data, and the details of each element are defined as follows.

- State space S: The status of AUV mobile collection is defined as
  (18) $$s_{t}=\left\{p_{a,t},\psi_{t},\Delta_{t},{\left\{x_{i,t}^{ac},x_{i,t}^{op},d_{i,t},\delta_{i,t}\right\}}_{i\in N}\right\},$$
  where $p_{a,t}$ and $\psi_{t}$ are the coordinates and sailing orientation of the AUV at time slot *t*, and its position can be obtained via ultra-short baseline (USBL) [44]. $\Delta_{t}$ is the difference between the remaining energy of the AUV and the AUV’s arrival at its final destination from its current position. $d_{i,t}$ records the Euclidean distance of the AUV to CH $c_{i}$. $x_{i,t}^{m}$ is the data collection indicator related to the data collection option. When the AUV arrives at the data collection point $c_{i}$, the AoI of node $c_{i}$ starts to be updated.
- Action space A: In state $s_{t}$, the action selection of the AUV is characterized by the target point $c_{i,t}\in N_{r}$ with the transmission option $m_{i,t}$, and the next target point $c_{j,t}\in N_{r}\backslash c_{i,t}$, where $N_{r}$ is the set of CHs that have not been collected. Then, the action performed by AUV at state $s_{t}$ can be expressed as
  (19) $$a=\left\{c_{i,t},m_{i,t},c_{j,t}|s_{t}\right\}.$$
- State transition probability P: $P\left(s_{t+1}\left|s_{t},a_{t}\right.\right)$ defines the transition probability from state $s_{t}$ to the next state $s_{t+1}$ under the action $a_{t}$, and $P\left(s_{t+1}\left|s_{t},a_{t}\right.\right)=1$ holds.
- Reward R: Applying action $a_{t}$ in state $s_{t}$, the AUV enters state $s_{t+1}$ and obtains an immediate reward $r\left(s_{t+1}\left|s_{t},a_{t}\right.\right)$. In the AUV-assisted multi-modal data collection scenario, the immediate reward $r_{t}$ can be expressed as
  (20) $$r_{t}=\left\{\begin{array}{cc} x_{i,t}^{ac}k_{1}e_{i}+x_{i,t}^{op}k_{2}e_{i}, & \mathrm{if}\;b_{i}=1\\ J, & \mathrm{if}\;\mathrm{done}\\ \eta_{i}\left(dis_{p_{a},p_{i}}+1\right), & \mathrm{otherwise} \end{array}\right.,$$
  where $k_{1}$, $k_{2}$ are constants and $k_{1}<k_{2}$ holds, and when the AUV has collected the data of CH $c_{i}$, the relevant reward is obtained according to the selected modem. $dis_{p_{a},p_{i}}$ is the Euclidean distance from the current position of the AUV to the target point. *J* denotes the reward at the end of the data collection process, including rewards for successful data collection and penalties for failure (e.g., exceeding maximum energy consumption and crossing boundaries).
  (21) $$J=\left\{\begin{array}{cc} r_{out}, & \mathrm{if}\;\;\Delta_{t}<0\;or\;p_{a}\notin \Omega \\ k_{3}-\frac{1}{N}\sum_{i=1}^{N}A_{i}, & \mathrm{if}\;p_{a}=p_{0}\;\mathrm{and}\;N_{r}=⌀ \end{array}\right.,$$
  where $k_{3}$ is a constant and Ω is the region in which the AUV can move within.
- Discount factor γ: γ∈[0,1] is the future reward discount factor.

### 5.2. Multi-Modal Steering Angle Optimization Algorithm

**Figure 2:**
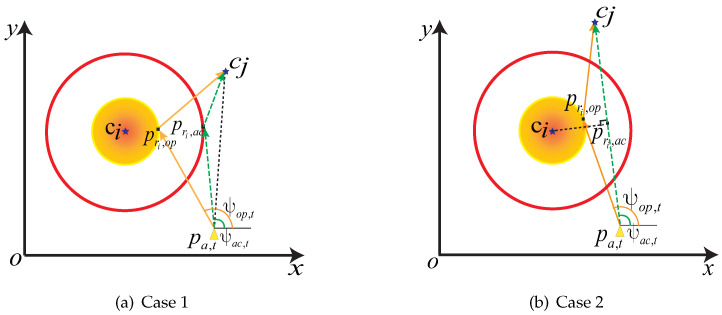
The AUV multi-modal steering angle diagram. (**a**) The trajectory of the AUV from the current coordinates $p_{a,t}$ towards the next target $c_{j}$ will not sail through the communication region $C_{m}$ of $c_{i}$. (**b**) The trajectory of the AUV from the current coordinates $p_{a,t}$ towards the next target $c_{j}$ will sail through the communication region $C_{m}$.

In the multi-modal data collection network, since the communication radius can reduce the navigation time of AUVs, we propose an MSAO algorithm to adjust the AUV heading under the maximum steering angle constraint. In MSAO, the steering angle of the AUV is calculated based on its position, the navigation target and the communication options. As shown in Figure 2, the yellow triangle indicates the position $p_{a,t}$ of the AUV at time slot *t*, the blue pentagram indicates the CHs that need to perform the data collection operation, the outer circle $C_{ac}$ and inner circle $C_{op}$ indicate the communication range of UAC and UOC, respectively. Let $c_{i}$ be the AUV’s current target CH and $c_{j}$ be the next target CH, the $p_{r_{i},m}$ indicates the target hover point when the AUV selects communication option $m=\left\{ac,op\right\}$, the $\psi_{m,t}$ indicates the angle of the AUV toward the target hover point $p_{r_{i},m}$ at time slot *t*. The goal is to obtain the point $p_{r_{i},m}$ such that the $∥p_{a,t}-p_{r_{i},m}∥+∥c_{j}-p_{r_{i},m}∥$ distance is shortest within the communication range $C_{m}$ of the communication options *m*. This problem is a classical pilgrimage problem in ancient castles, and hence, an approximate solution of $p_{r_{i},m}=\left(x_{r_{i}},y_{r_{i}}\right)$ can be obtained by the following equation [45].

(22) $$-\varsigma_{1}\sqrt{1-y^{2}}+\varsigma_{2}y=0,$$

where $\varsigma_{1}=\frac{x_{i}-x_{j}}{d_{m}d_{ij}}-\frac{x_{i}-x_{a,t}}{d_{m}d_{ai,t}}$, $\varsigma_{2}=\frac{y_{i}-y_{a,t}}{d_{m}d_{ai,t}}-\frac{y_{i}-y_{j}}{d_{m}d_{ij}}$, $y=\frac{y_{i}-y_{r_{i}}}{d_{m}}$, $d_{ai,t}$ and $d_{ij}$ denote the distance of the AUV from the target at time slot *t* and the distance of the current target CH $c_{i}$ from the next target CH $c_{j}$, respectively. Then, the steering angle of the AUV at time slot *t* can be expressed as    

(23) $$\Psi_{m,t}=\left\{\begin{array}{c} \min\left(\psi_{m,t}-\psi_{t},\psi_{\max}\right),\psi_{m,t}\geq \psi_{t}\\ \max\left(\psi_{m,t}-\psi_{t},-\psi_{\max}\right),\psi_{m,t}<\psi_{t} \end{array}\right.,$$

where $\psi_{\max}$ is the maximum steering angle allowed by the AUV. Then, depending on the target location and the communication option, the steering angle of the AUV can be adjusted in the following two cases.

- Case 1: The AUV is not through the region $C_{m}$ from the current position $p_{a,t}$ to the next target collection point $c_{j}$; i.e., the distance $d_{seg_{i}}$ from point $c_{i}$ to the segment $\bar{p_{a,t}c_{j}}$ is greater than the UAC radius. As shown in Figure 2a, after determining the communication option, the points $p_{r_{i},ac}$ (or $p_{r_{i},op}$) are obtained in circle $C_{ac}$ (or $C_{op}$) to minimize the length of the AUV trajectory. For example, when the CH $c_{i}$, $c_{j}$ and acoustic modem are selected, the AUV hover position $p_{r_{i},ac}=\left(x_{r_{i}},y_{r_{i}}\right)$ for data collection and the steering angle $\Psi_{ac,t}$ can be calculated by (22) and (23), respectively. Similarly, when m=op holds, the data collection hover point $p_{r_{i},op}$ and the steering angle $\Psi_{op,t}$ can be obtained using the same approach.
- Case 2: The trajectory of the AUV from the current coordinate $p_{a,t}$ to the next target CH $c_{j}$ sails through the communication region $C_{m}$ of $c_{i}$. If the AUV crosses the UAC area $C_{ac}$ without crossing the communication area $C_{op}$, $d_{seg_{i}}$ becomes shorter than $d_{ac}$ but greater than $d_{op}$. As shown in Figure 2b, the data collection hover point of the AUV is the vertical foot $p_{r_{i},ac}$ from $c_{i}$ to segment $\bar{p_{a,t}c_{j}}$ if UAC is selected as the communication option. Then, the steering angle of the AUV can be obtained by (23). If the selected communication option is UOC, the data collection point and steering angle are calculated following the method in Case 1. Furthermore, if $d_{seg_{i}}$ is less than $d_{op}$, i.e., the AUV crosses the UOC range of $c_{i}$, then UOC is selected directly as the communication option. This is due to the superiority of UOC over UAC in terms of energy consumption and transmission time for the same AUV trajectory. The data collection hover point and steering angle of the AUV are similar to the method in Case 2.

Based on the above discussion, we obtain the MSAO algorithm that is shown in Algorithm 1.
**Algorithm 1** Proposed MSAO Algorithm**Require:** Coordinate of the AUV $p_{a,t}$, coordinates of the current target CH $c_{i}$, coordinates of the next target CH $c_{j}$, UAC communication radius $d_{ac}$ and UOC communication radius $d_{op}$.  1:**if**$d_{seg_{i}}>d_{ac}$ and m=ac **then**  2:    Calculate the data collection hover position $p_{c_{i},ac}$ by (22).  3:    Calculate steering angle $\Psi_{ac,t}$ by (23).  4:**else if**$d_{seg_{i}}\leq d_{ac}$ and $d_{seg_{i}}>d_{op}$ and m=ac **then**  5:    The data collection hover position is the vertical foot $p_{r_{i},ac}$     from $c_{i}$ to the segment $\bar{p_{a,t}c_{j}}$.  6:    Calculate steering angle $\Psi_{ac,t}$ by (23).  7:**else if**$d_{seg_{i}}>d_{op}$ and m=op **then**  8:    Calculate the data collection hover position $p_{r_{i},op}$ by (22).  9:    Calculate steering angle $\Psi_{op,t}$ by (23).10:**else if**$d_{seg_{i}}\leq d_{op}$**then**11:    The data collection hover position is the vertical foot $p_{r_{i},op}$     from $c_{i}$ to the segment $\bar{p_{a,t}c_{j}}$.12:    Calculate steering angle $\Psi_{op,t}$ by (23).13:**end if****Ensure:** The steering angle of the AUV: $\Psi_{m,t}$.

### 5.3. DRL-Based Multi-Modal Path Planning Scheme

Due to the uncertainty of reference access points and node data arrivals, the locations of AUV and the AoI of collected data are inherently random, which leads to a proliferation of state space dimensions. In comparison, DRL can handle extremely large state space by estimating the *Q* values of states *s* and actions *a* through neural networks [45,46]. The training framework of DQN includes a current Q-network and a target Q-network. In order to balance experience and exploration of the unknown, the agent at state $s_{t}$ selects the action $a_{t}$ to be performed by the ϵ-greedy algorithm [47].

(24) $$a_{t}=\left\{\begin{array}{cc} random\;a\in A, & \mathrm{with}\;\mathrm{probability}\;\varepsilon \\ \arg\max_{a_{t}}Q\left(s_{t},a_{t};\theta \right), & \mathrm{with}\;\mathrm{probability}\;1-\varepsilon \end{array}\right..$$

Immediately after adjusting the navigation angle and execution of action $a_{t}$, the AUV receives reward $r_{t}$ and the data acquisition network moves to the next state $s_{t+1}$. Aiming to reduce the correlation between the online Q-network samples, an experience replay B is used to store historical experience samples $\left(s_{t},a_{t},r_{t},s_{t+1}\right)$. At each training step, a small batch of randomly selected empirical samples $\Phi_{b}$ from the experience replay is used to update the parameters of the online Q-network. In addition, we denote the parameters of DQN as θ, and the parameters of the online Q-network are determined by minimizing the loss function.

(25) $$L\left(\theta \right)=E_{\Phi_{b}}\left[\delta {\left(s,a\right)}^{2}\right],$$

where $\delta \left(s,a\right)=y_{t}-Q\left(s_{t},a_{t}|\theta \right)$ is the temporal difference, and $y_{t}$ is the target Q-value, which can be calculated by

(26) $$y_{t}=r_{t}+\gamma \max_{a_{t+1}}Q\left(s_{t+1},a_{t+1}|\theta^{-}\right),$$

where $\theta^{-}$ denoted the parameter of the target Q-network. Then, the weight of the current network θ is updated by the following formula.

(27) $$\theta =\theta +\frac{\alpha }{\Phi_{b}}\sum_{t=1}^{\Phi_{b}}\delta (s,a)\nabla_{\theta }Q\left(s_{t},a_{t},\theta \right).$$

The proposed AUV-assisted data collection algorithm is shown in Algorithm 2. The algorithm starts by initializing all neural networks as well as the replay buffer B. The training iterates over *E* episodes, and the environment is initialized in each episode by observing the distribution of CHs. The action is first obtained according to the ϵ-greedy policy, which is followed by inputting the action to Algorithm 1 to obtain the steering angle. Then, the AUV moves to the next state $s_{t+1}$ and receives an immediate reward $r_{t}$. After storing the transition tuple $\left(s_{t},a_{t},r_{t},s_{t+1}\right)$ in experience replay B, a randomly selected sample of $\Phi_{b}$ is utilized to learn the current network *Q*, and it updates the weights of the current network θ and that of the target network $\theta^{-}$. Then, $c_{i}$ is removed from $N_{r}$ if the current state is able to collect the data of $c_{i}$, and the current loop is terminated when $N_{r}=\emptyset$ holds.
**Algorithm 2** DRL-Based Multi-Modal Data Collection Algorithm  1:**Input:** Initialize the constants $k_{1}$, $k_{2}$ and $k_{3}$, maximum number of training sets *E*, reward discount factor γ, learning rate $l_{r}$, experience replay B, minimum batch $\Phi_{b}$, exploration probability ϵ, and update step χ;  2:Initialize the current network $Q(s_{t},a_{t},\theta )$ with weights θ and the target network $Q(s_{t},a_{t},\theta^{-})$ with weights $\theta^{-}$.  3:**for**episode=1,⋯,E**do**  4:    **for** t=1,⋯,T **do**  5:        Initialize the data collection network environment and observe the initial state $s_{t}$.  6:        Select a random action $a_{t}$ according to the ϵ-greedy algorithm.  7:        Determine the AUV steering angle with Algorithm 1.  8:        Execute action $a_{t}$ and observe the reward $r_{t}$ and the next state $s_{t+1}$.  9:        Store experience $\left(s_{t},a_{t},r_{t},s_{t+1}\right)$ in experience replay B.10:        Sample a random mini-batch of $\Phi_{b}$ experiences from B.11:        Calculate the target value $y_{t}$ by (26).12:        Update the current network weights θ by (27).13:        Update the weights of the target network $\theta^{-}=\theta$ every χ steps.14:        **if** $s_{t+1}$ is the collection stop $n_{i}$ **then**15:           Remove the CH $c_{i}$ from $N_{r}$.16:        **end if**17:        Terminate the episode if $N_{r}=\emptyset$ holds.18:    **end for**19:**end for**20:**Output:** The AUV trajectory $p_{a,t}$ and the AoI $A_{i}$.

## 6. Results and Discussion

In this section, we conduct extensive simulations to verify the effectiveness of the proposed scheme. The simulation setup and numerical performance results are given as follows.

### 6.1. Simulation Setup

To evaluate the proposed scheme, we assume that there are 50 sensor nodes uniformly distributed in an 800 m × 800 m square target area. After CHs are designated, data fusion and data compression are performed by CHs. It is assumed that the data types collected and transmitted by the normal sensor nodes are text, records and images, and the amount of data pooled by the CHs is set to be between 10 and 300 packets, with the size of each packet 1024 bits. The AUV starts from the start point $p_{0}=$ (50, 120) with an initial orientation angle $\psi_{m,0}=0^{\circ }$ and returns to $p_{0}$ after collecting data from all the CHs.

To evaluate the performance of the algorithm, a python 3.8 simulation environment was chosen. The target Q-network and the current Q-network are two-layer fully connected networks with 256 neurons per layer, and we use the ReLU function as the activation function to train both networks using the Adam optimizer. Other simulation parameters and their specific values are provided in Table 1.

For the sake of performance comparison, the benchmark algorithms are provided as follows.

- Single Acoustic: The AUV exchanges data utilizing acoustic waves during data collection, and the hovering positions are determined by the UAC radius during the selection process of the steering angle. The AUV trajectories are learned using the DQN algorithm.
- Single Optical: The AUV can exchange data only by selecting optical waves and calculating the AUV hovering locations by means of the UOC radius. The DQN algorithm is used to learn the AUV trajectory.
- Energy Greedy: The AUV performs steering Algorithm 1 and then greedily selects the nodes with the shortest path length in the data collection sequence.

### 6.2. The Convergence Performance

**Figure 3:**
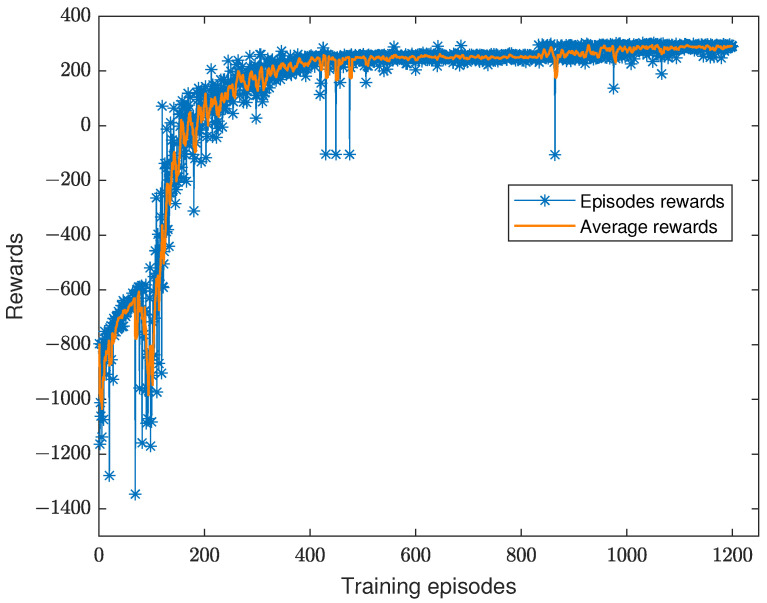
Convergence performance of the DQN-based data collection algorithm.

To demonstrate the convergence of the AoI optimization algorithm for AUV data collection, in Figure 3, we show the variation of the cumulative reward, where the X-axis represents the number of iterations trained and the Y-axis presents the cumulative reward. It can be seen that in the early stages of training, the cumulative reward values are very low due to the high chance of ϵ-greedy random exploration. As the training period continues to increase, the reward value gradually increases and stabilizes.

### 6.3. Impact of the AUV Velocity on Performance

**Figure 4:**
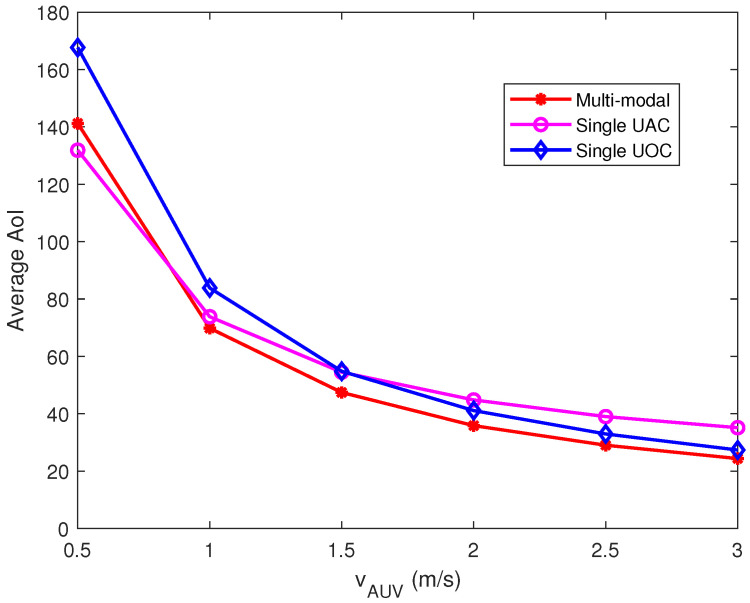
Effect of AUV velocity on AoI of collected data.

To explore the effect of AUV velocity on the average AoI, we simulated the average AoI performance of the collected data with data arrival rate λ = 20 and 300 Kbits. The experimental results are shown in Figure 4, where it can be observed that the average AoI of the data collected by AUV gradually decreases with increasing AUV velocity. The weighted average AoI of the single UAC is lower than that of the multi-modal and single UOC when the AUV speed is 0.5 m/s. With the increase of the AUV velocity, the average AoI of the multi-modal data collection scheme is better than that of the single communication option.

The primary reason for this performance is that the AUV travels slower and increases travel time, whereas the long-distance data collection via the single UAC is able to reduce the travel time of the AUV, which mitigates the increase in AoI. As the AUV velocity increases, the effect of AUV travel time on AoI is weakened, which makes the weight of data transmission time increase for AoI; thus, the UOC scheme outperforms the UAC scheme at higher AUV velocities. The multi-modal data collection scheme selects the best communication option according to the data characteristics, AUV navigation time and data transmission time so that the overall performance is better than the single-modal scheme. Furthermore, the average AoI of our proposed multi-modal data collection scheme at the AUV velocity of 0.5 m/s is inferior to that of the single UAC scheme; this is because the multi-modal scheme not only considers AoI but also focuses on data collection energy consumption, so it sacrifices some AoI performance to reduce CHs energy consumption.

**Figure 5:**
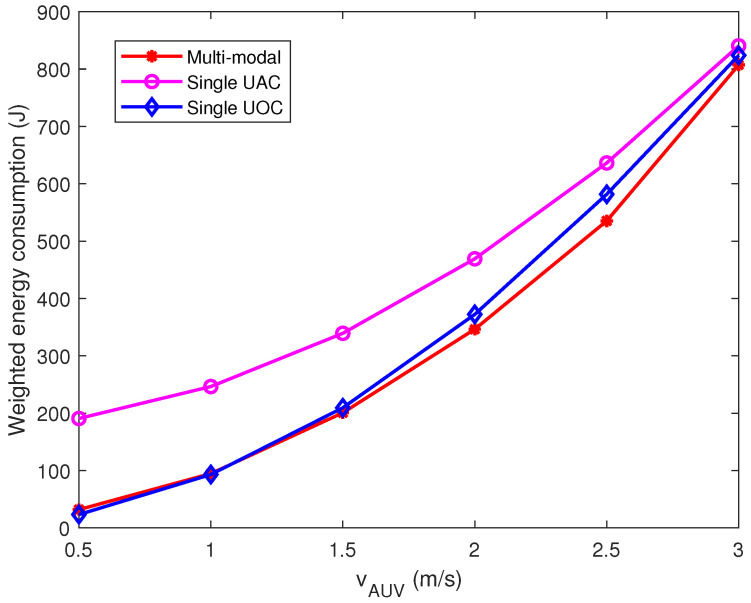
Effect of AUV velocity on the weighted energy consumption of the task.

Under the parametric conditions of Figure 4, we analyzed the effect of AUV velocity on the energy consumption of data collection. Considering that the energy consumption of CHs is irreversible, we pay more attention to the energy consumption of data transmission, and therefore, we set ϖ = 0.03. As shown in Figure 5, it can be observed that the weighted energy consumption of the single UAC scheme always remains at the highest level owing to the high weight of data transmission energy consumption. The single UOC scheme performs well in terms of CHs data transmission energy consumption, and hence, the weighted energy consumption is better than the single UAC scheme. The multi-modal scheme is able to reduce both AUV energy consumption and CHs data transmission energy consumption by jointly deciding on the best communication option based on packet size and path length. Furthermore, with the increasing AUV velocity, the weighted energy consumption of the three schemes will be convergent, since the AUV power increases geometrically with the velocity of travel.

### 6.4. Impact of the Data Arrival Rate on Performance

**Figure 6:**
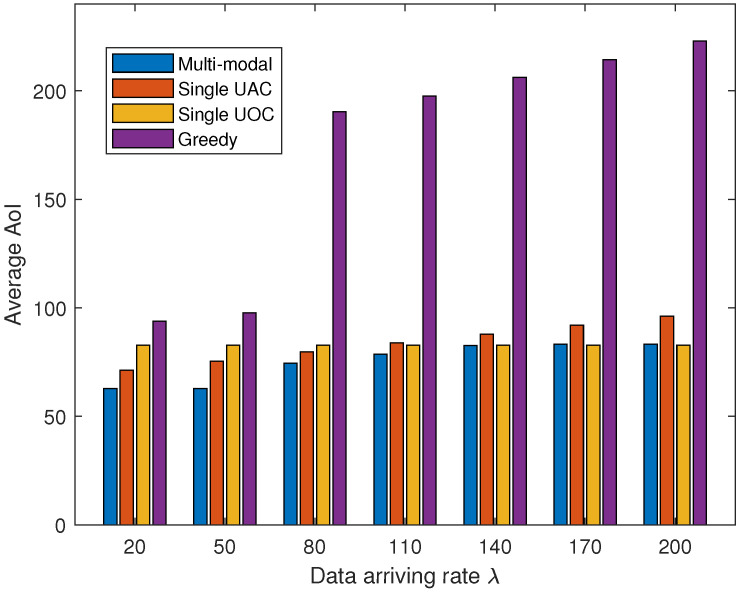
The average AoI of collected data with the increasing data arriving rate.

Figure 6 shows the effect of different data arrival rates on the weighted average AoI. The velocity of the AUV is set to 1 m/s, and the length of each time slot is set to 6 s. It can be observed from the figure that at lower data arrival rates, the single UAC is superior to the single UOC since the acoustic waves can be deployed for long-range data transmission, which significantly saves the travel time of the AUV. As the data arrival rate increases, the weight of data transmission time on AoI improves, which results in the single UOC scheme being superior to the single UAC scheme in terms of AoI. In addition, the greedy algorithm performs poorly in weighted average AoI as it greedily selects the closest visit location ignoring data importance and AoI. Our proposed multi-modal data collection scheme outperforms the other three schemes for different data arrival rates and is near the single UOC performance when the data size is over 140 Kbits. The main reason for this phenomenon is that the multi-modal scheme selects acoustic communication to reduce the sailing time when the data size is small and optical communication for fast data transmission when the data size is large, and thus, it can adapt to different data conditions and achieve a relatively low weighted average AoI.

**Figure 7:**
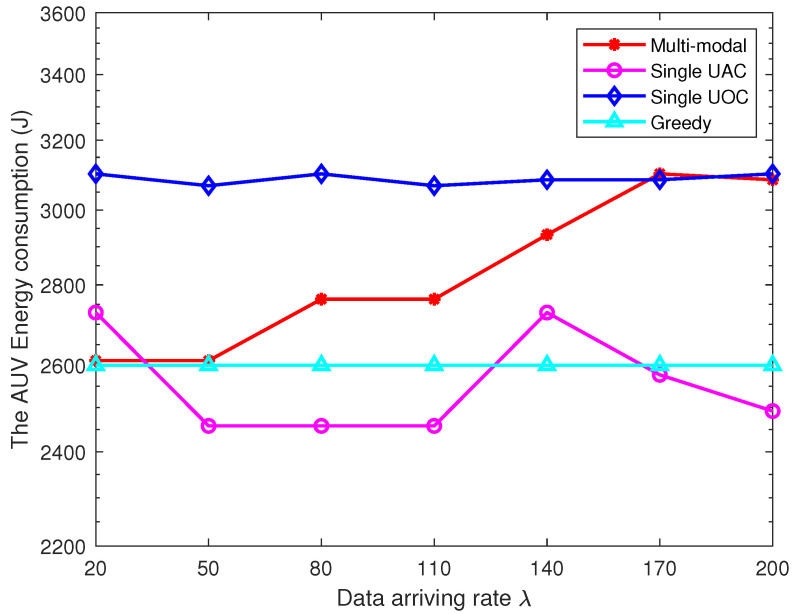
Performance comparison in terms of AUV energy consumption.

To verify the superiority of the proposed multi-modal data collection algorithm in terms of energy consumption for data collection, we compare the transmission energy consumption of CHs and AUV energy consumption for different data sizes. In this study, we set the AUV velocity to 1 m/s and the data size to 20–200 Kbits, and the experimental results are shown in Table 2 and Figure 7. It is observed that the average data transmission energy consumption of CHs under a single UOC approach is the smallest, and the AUV energy consumption is the highest. The energy consumption of CHs is the highest for the single UAC and greedy approaches, but the AUV energy consumption is kept at a low level. In the multi-modal scheme, the energy consumption of CH increases and then decreases with the increasing data size, and the AUV energy consumption gradually increases, but the overall energy consumption remains at a low level.

The reason for such a phenomenon can be explained as follows. The single UOC requires the AUV to travel to the immediate vicinity of the node, which increases the energy consumption of the AUV for navigation. Fortunately, due to the low energy consumption and high bandwidth of the optical modems, the energy consumption of the CHs owing to the data transmission is low. Similarly, the single UAC and greedy algorithm allow the AUV to collect data over longer distances using acoustic waves, which greatly saves AUV energy consumption. However, with the increasing data size, the low bandwidth and high energy consumption of the UAC make the energy consumption of the CHs significant. In the multi-modal scheme, the AUV selects the UAC to collect data when the data size is small, keeping the transmission energy consumption of the CHs low while reducing the mobile energy consumption. When the data size is larger than 140 Kbits, the multi-modal scheme switches to strictly optical communication mode to reduce the excessive energy consumption of the CHs in order to extend the lifetime of the UWSN. Note that our proposed multi-modal data collection scheme has an excellent performance in the face of diversified data, and when the data size of each CHs is large (or small), the multi-modal scheme will become a strict UOC (or UAC) scheme.

**Figure 8:**
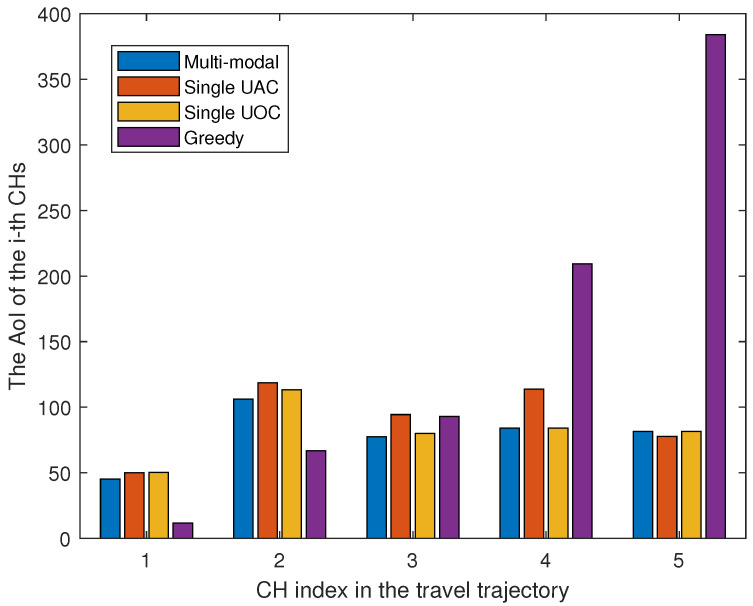
AoI for different paths when the number of CHs is 5.

In Figure 8, we show the weights of AoI for each CHs under different schemes. The results show that the greedy scheme has the maximum AoI value for CH index = 5 and the lowest AoI value for index = 1. This is because the greedy algorithm ignores the effect of data importance when selecting the nearest nodes to visit, resulting in a large data AoI for the first visited node. The other three schemes use reinforcement learning methods to select the best node access order based on the importance of the data, which avoids the extreme cases of AoI values. Furthermore, the multi-modal scheme flexibly selects the communication options based on the data size and importance of the nodes, and hence, its performance is better compared to the other two single-modal schemes. It is worth noting that we neglected the specific details of light alignment and the time consumed during data collection, which result in a seemingly promising AoI performance for the single UOC. In future work, we will consider more details of underwater optical communication.

## 7. Conclusions

In this paper, we proposed an AUV-assisted multi-modal data collection scheme which provides timely and reliable data collection by utilizing underwater acoustic and optical communication technologies in an adaptive manner. The trajectory planning problem is formulated as a mixed integer nonlinear problem to minimize the weighted average AoI and energy consumption, and the data collection problem is formulated as an MDP considering data importance, packet size, and data collection options. We then developed a DQN-based learning algorithm to determine the optimal strategy. In addition, an AUV multi-modal corner optimization algorithm is proposed to reduce the energy consumption of AUV navigation. Through numerical simulations, we showed that our proposed algorithm has convergence capability as well as verified that the AUV path-planning algorithm has excellent performance which can effectively reduce the AoI and energy consumption of collected data.

## Figures and Tables

**Table 1:** Simulation parameters.

Parameters	Description	Value (Unit)
*f*	Carrier frequency	35 (kHz)
Δf	Bandwidth	2 (kHz)
*k*	Propagation loss	1.5
$SNR_{\min}^{ac}$	UAC minimum SNR	3 (dB)
$R_{ac}$	UAC data rate	3.16 (kbps)
$R_{op}$	UOC data rate	0.5 (Gbps)
θ	Half of the transmitter beamwidth	3 ${(}^{\circ })$
*c*	Extinction coefficient	0.18 (m${}^{-1}$)
ζ	Turbidity of water quality	0.05
$D_{r}$	Aperture diameter	0.25 (m)
$SNR_{\min}^{op}$	UOC minimum SNR	3 (dB)
NEP	Noise equivalent power	1 (mW)
$P_{rec}^{op}$	Average transmitted power	0.01 (mW)
ρ	Density of water	997 (Kg/m${}^{3}$)
$\eta_{p}$	Efficiency of the AUV propulsion system	100%
$C_{D}$	Drag coefficient	0.0064
$A_{s}$	Wetted surface area	0.8856 (m${}^{2}$)
B	Experience replay buffer sizer	500,000
$\Phi_{b}$	Mini-batch size	256
γ	Reward discount factor	0.95
χ	Update step	1000

**Table 2:** Average data transmission energy consumption of CHs versus the data arriving rate.

Collected Data (Kbits)	Single UAC (mJ)	Single UOC (mJ)	Greedy (mJ)	Multi-Modal (mJ)
20	6659.29	0.02	5650.31	5112.19
50	15,403.83	0.05	12,780.47	3968.71
80	24,081.09	0.08	20,852.35	5583.11
110	32,623.83	0.11	28,789.69	7668.36
140	41,099.29	0.14	379,37.81	0.14
170	49,103.91	0.16	46,346.02	0.16
200	52,399.92	0.19	55,359.61	0.19

## Data Availability

Not applicable.

## References

[1] Jahanbakht M., Xiang W., Hanzo L., Azghadi M.R. (2021). Internet of underwater things and big marine data analytics—A comprehensive survey. IEEE Commun. Surveys Tuts..

[2] Wei X., Guo H., Wang X., Wang X., Qiu M. (2021). Reliable data collection techniques in underwater wireless sensor networks: A survey. IEEE Commun. Surveys Tuts..

[3] Luo H., Wu K., Ruby R., Liang Y., Guo Z., Ni L.M. (2018). Software-defined architectures and technologies for underwater wireless sensor networks: A survey. IEEE Commun. Surveys Tuts..

[4] Luo H., Wang J., Bu F., Ruby R., Wu K., Guo Z. (2022). Recent progress of air/water cross-boundary communications for underwater sensor networks: A review. IEEE Sens. J..

[5] Fang Z., Wang J., Jiang C., Zhang Q., Ren Y. (2021). AoI-inspired collaborative information collection for AUV-assisted internet of underwater things. IEEE Internet Things J..

[6] Jawhar I., Mohamed N., Al-Jaroodi J., Zhang S. (2018). An architecture for using autonomous underwater vehicles in wireless sensor networks for underwater pipeline monitoring. IEEE Trans. Ind. Informat..

[7] Mahmoodi K.A., Uysal M. AUV Trajectory Optimization for an Optical Underwater Sensor Network in the Presence of Ocean Currents. Proceedings of the 2021 IEEE International Black Sea Conference on Communications and Networking (BlackSeaCom).

[8] Wang X., Luo H., Yang Y., Ruby R., Wu K. Underwater Real-time Video Transmission via Optical Channels with Swarms of AUVs. Proceedings of the 2021 IEEE 27th International Conference on Parallel and Distributed Systems (ICPADS).

[9] Wang J., Li J., Yan S., Shi W., Yang X., Guo Y., Gulliver T.A. (2020). A novel underwater acoustic signal denoising algorithm for Gaussian/non-Gaussian impulsive noise. IEEE Trans. Veh. Technol..

[10] Akhoundi F., Jamali M.V., Hassan N.B., Beyranvand H., Minoofar A., Salehi J.A. (2016). Cellular underwater wireless optical CDMA network: Potentials and challenges. IEEE Access.

[11] Wang J., Luo H., Ruby R., Liu J., Guo K., Wu K. Reliable Water-Air Direct Wireless Communication: Kalman Filter-Assisted Deep Reinforcement Learning Approach. Proceedings of the 2022 IEEE 47th Conference on Local Computer Networks (LCN).

[12] Luo H., Xie X., Han G., Ruby R., Hong F., Liang Y. (2019). Multimodal acoustic-RF adaptive routing protocols for underwater wireless sensor networks. IEEE Access.

[13] Zhao Z., Liu C., Qu W., Yu T. An Energy Efficiency Multi-Level Transmission Strategy based on underwater multimodal communication in UWSNs. Proceedings of the IEEE INFOCOM 2020-IEEE Conference on Computer Communications.

[14] Gauni S., Manimegalai C., Krishnan K.M., Shreeram V., Arvind V., Srinivas T. (2021). Design and analysis of co-operative acoustic and optical hybrid communication for underwater communication. Wireless Pers. Commun..

[15] Yu T., Liu C., Qu W., Zhao Z. OD-PPS: An On-Demand Path Planning Scheme for Maximizing Data Completeness in Multi-modal UWSNs. Proceedings of the International Conference on Wireless Algorithms, Systems, and Applications.

[16] Gjanci P., Petrioli C., Basagni S., Phillips C.A., Bölöni L., Turgut D. (2018). Path finding for maximum value of information in multi-modal underwater wireless sensor networks. IEEE Trans. Mobile Comput..

[17] Hu Y., Zheng Y., Liu H., Wang Z., Mao Y., Han H. Mobile sink path planning research for underwater heterogeneous sensor network. Proceedings of the 2018 Chinese Control And Decision Conference (CCDC).

[18] Han S., Noh Y., Liang R., Chen R., Cheng Y.J., Gerla M. (2014). Evaluation of underwater optical-acoustic hybrid network. China Commun..

[19] Sun Y., Uysal-Biyikoglu E., Yates R.D., Koksal C.E., Shroff N.B. (2017). Update or wait: How to keep your data fresh. IEEE Trans. Inf. Theory.

[20] Talak R., Karaman S., Modiano E. Optimizing information freshness in wireless networks under general interference constraints. Proceedings of the Eighteenth ACM International Symposium on Mobile Ad Hoc Networking and Computing.

[21] Yi M., Wang X., Liu J., Zhang Y., Bai B. Deep reinforcement learning for fresh data collection in UAV-assisted IoT networks. Proceedings of the IEEE INFOCOM 2020—IEEE Conference on Computer Communications Workshops (INFOCOM WKSHPS).

[22] Basagni S., Di Valerio V., Gjanci P., Petrioli C. (2019). MARLIN-Q: Multi-modal communications for reliable and low-latency underwater data delivery. Ad Hoc Netw..

[23] Shen Z., Yin H., Jing L., Liang Y., Wang J. (2021). A Cooperative Routing Protocol Based on Q-Learning for Underwater Optical-Acoustic Hybrid Wireless Sensor Networks. IEEE Sens. J..

[24] Júnior E.P.C., Vieira L.F., Vieira M.A. (2020). CAPTAIN: A data collection algorithm for underwater optical-acoustic sensor networks. Comput. Netw..

[25] Luo H., Xu Z., Wang J., Yang Y., Ruby R., Wu K. (2022). Reinforcement Learning-Based Adaptive Switching Scheme for Hybrid Optical-Acoustic AUV Mobile Network. Wirel. Commun. Mob. Com..

[26] Liu Z., Meng X., Liu Y., Yang Y., Wang Y. (2021). AUV-Aided Hybrid Data Collection Scheme Based on Value of Information for Internet of Underwater Things. IEEE Internet Things J..

[27] Duan R., Du J., Jiang C., Ren Y. (2020). Value-based hierarchical information collection for AUV-enabled internet of underwater things. IEEE Internet Things J..

[28] Fang Z., Wang J., Jiang C., Wang X., Ren Y. (2022). Average Peak Age of Information in Underwater Information Collection with Sleep-scheduling. IEEE Trans. Veh. Technol..

[29] Khan M.T.R., Jembre Y.Z., Ahmed S.H., Seo J., Kim D. Data freshness based AUV path planning for UWSN in the internet of underwater things. Proceedings of the 2019 IEEE Global Communications Conference (GLOBECOM).

[30] Al-Habob A.A., Dobre O.A., Poor H.V. (2021). Age-optimal information gathering in linear underwater networks: A deep reinforcement learning approach. IEEE Trans. Veh. Technol..

[31] Wu T., Wen P., Tang S. (2022). Optimal scheduling strategy of AUV based on importance and age of information. Wirel. Netw..

[32] Huang M., Zhang K., Zeng Z., Wang T., Liu Y. (2020). An AUV-assisted data gathering scheme based on clustering and matrix completion for smart ocean. IEEE Internet Things J..

[33] Cheng W., Teymorian A.Y., Ma L., Cheng X., Lu X., Lu Z. Underwater localization in sparse 3D acoustic sensor networks. Proceedings of the IEEE INFOCOM 2008—The 27th Conference on Computer Communications.

[34] Stojanovic M., Preisig J. (2009). Underwater acoustic communication channels: Propagation models and statistical characterization. IEEE Commun. Mag..

[35] Brekhovskikh L.M., Lysanov Y.P., Beyer R.T. (2003). Fundamentals of Ocean Acoustics.

[36] Stojanovic M. (2007). On the relationship between capacity and distance in an underwater acoustic communication channel. Proceedings of the ACM SIGMOBILE Mobile Computing and Communications Review.

[37] Elamassie M., Miramirkhani F., Uysal M. (2018). Performance characterization of underwater visible light communication. IEEE Trans. Commun..

[38] Giles J.W., Bankman I.N. Underwater optical communications systems. Part 2: Basic design considerations. Proceedings of the MILCOM 2005–2005 IEEE Military Communications Conference.

[39] Steinvall O. (2009). Laser system range calculations and the Lambert W function. Appl. Opt..

[40] Farr N., Chave A., Freitag L., Preisig J., White S., Yoerger D., Titterton P. Optical modem technology for seafloor observatories. Proceedings of the OCEANS 2005 MTS/IEEE.

[41] Liu J., Wang X., Bai B., Dai H. Age-optimal trajectory planning for UAV-assisted data collection. Proceedings of the IEEE INFOCOM 2018-IEEE Conference on Computer Communications Workshops (INFOCOM WKSHPS).

[42] Phillips A., Haroutunian M., Murphy A.J., Boyd S., Blake J., Griffiths G. (2017). Understanding the power requirements of autonomous underwater systems, Part I: An analytical model for optimum swimming speeds and cost of transport. Ocean Eng..

[43] Furlong M.E., McPhail S.D., Stevenson P. A concept design for an ultra-long-range survey class AUV. Proceedings of the OCEANS 2007-Europe.

[44] Rigby P., Pizarro O., Williams S.B. Towards geo-referenced AUV navigation through fusion of USBL and DVL measurements. Proceedings of the OCEANS 2006.

[45] Su N., Wang J.B., Zeng C., Zhang H., Lin M., Li G.Y. (2022). Unmanned-Surface-Vehicle-Aided Maritime Data Collection Using Deep Reinforcement Learning. IEEE Internet Things J..

[46] Mnih V., Kavukcuoglu K., Silver D., Rusu A.A., Veness J., Bellemare M.G., Graves A., Riedmiller M., Fidjeland A.K., Ostrovski G. (2015). Human-level control through deep reinforcement learning. Nature.

[47] dos Santos Mignon A., da Rocha R.L.d.A. (2017). An adaptive implementation of *ϵ*-greedy in reinforcement learning. Procedia Comput. Sci..

